# Comorbidity and the diagnosis of symptomatic-but-as-yet-undiagnosed cancer

**DOI:** 10.3399/bjgp20X712193

**Published:** 2020-08-19

**Authors:** Cristina Renzi, Georgios Lyratzopoulos

**Affiliations:** Epidemiology of Cancer Healthcare and Outcomes (ECHO) Group, University College London, London, UK.; ECHO Group, University College London, London, UK.

Multimorbidity is the norm of modern medicine. We know that multimorbidity affects healthcare use and outcomes,^1^ but we don’t know how the presence of pre-existing conditions can influence the diagnosis of new illness. Cancer typically affects older patients, and presents with symptoms of relatively low specificity that are shared between different conditions. Timely diagnosis is important,^2^^,^^3^ but missed or delayed diagnoses are common.^4^ Might the presence of chronic conditions help us understand why the diagnosis of patients with symptomatic-but-as-yet-undiagnosed cancer is delayed? Carney *et al*, in this issue of the *BJGP*, shed light on this complex question in the context of the diagnosis of bladder cancer.^5^

## MECHANISMS BY WHICH COMORBIDITY MAY INFLUENCE THE DIAGNOSIS OF CANCER

Pre-existing chronic conditions may act as ‘competing demands’ that prevent the investigation of new presenting symptoms or offer ‘alternative explanations’ for them.^6^^,^^7^ For cancers of specific organs or systems, different morbidities can be categorised into those with unrelated symptomatology and those with shared symptoms. For patients with symptomatic-but-as-yet-undiagnosed bladder cancer, Carney *et al* consider that previous urinary tract infections, prostatitis, and nephrolithiasis can offer alternative explanations for their symptoms. Similar considerations may apply to other ‘dyads’ of specific cancer sites and chronic conditions, for example, lung cancer and chronic obstructive pulmonary disease, or colorectal cancer and inflammatory bowel disease.

It can be difficult to distinguish between a genuinely pre-existing morbidity and a cancer initially misdiagnosed as a chronic condition. The study by Carney *et al* highlights that when using electronic health records, careful consideration is needed regarding the time when morbidities were first recorded, ideally handling the months before the diagnosis of cancer differently from earlier periods. Such analyses can also indicate possible opportunities for earlier diagnosis in subgroups of comorbid patients who are at greater risk of repeat presentations with cancer-related symptoms before an emergency cancer diagnosis.^8^

Chronic conditions offering ‘alternative explanations’ were found to be associated with a higher risk of diagnosis of advanced stage bladder cancer.^5^ The study by Carney *et al* did not directly examine the ‘surveillance effect’ hypothesis, where chronic disease monitoring for underlying morbidities might lead to earlier detection of new illness through more frequent contacts with healthcare professionals.^6^^,^^8^^–^^10^ However, some of the study findings would be consistent with such a mechanism.

It is important to explore if the association between morbidity and stage at diagnosis is causal. And, if so, how it may be mediated through management decisions and intervals to testing. Given the possible mechanisms involved and that morbidity-specific effects might vary in their size and direction by cancer site, symptoms, and patient characteristics, future studies will require large patient numbers.

The study by Carney *et al* represents a welcome addition to the evidence base.^6^^–^^8^^,^^11^ Most previous studies lacked a clear hypothesis on responsible mechanisms and did not consider the type of presenting symptoms or underlying conditions. They often defined morbidity only using summary measures, such as the Charlson Comorbidity Index, and used secondary care data, which will underestimate the true breadth and prevalence of certain morbidities.^6^

## FUTURE DIRECTIONS

Focusing on specific cancer sites and chronic conditions helps illuminate the relationship between morbidity, the diagnostic process, and its outcomes. Because many patients will have multiple chronic conditions, future research should explore if effects vary across different morbidity clusters.^1^ Encompassing prescription history into such inquiries is also important, as certain pharmacological treatments for managing chronic diseases, such as aspirin,^12^ can influence cancer incidence, and possibly cancer aggressiveness. Large longitudinal studies based on electronic health records offer promise for shedding light in this complex area.

Examining different steps along the diagnostic pathway is essential for developing appropriate interventions, bearing in mind that morbidities can influence timely access to appropriate investigations even after referral to hospital services.^13^ Cognitive and emotional factors may influence decisions on use of invasive investigations in patients with serious cardiac or respiratory morbidities. Comorbidities may also affect patients’ symptom appraisal and help-seeking behaviour.^9^^,^^10^

When assessing trade-offs between risks and benefits, preferences and tolerance of uncertainty, by both patients and doctors, can be important; therefore, greater understanding of shared decision- making in the management of patients with multimorbidity is needed. Risk-stratification tools that take chronic morbidities and their treatments into account can be developed, enhancing currently available generic instruments, to support clinicians in the decision-making process when evaluating the possibility of cancer in symptomatic patients with multiple morbidities.
